# Effects of scorpion venom heat-resistant peptide on the hippocampal neurons of kainic acid-induced epileptic rats

**DOI:** 10.1590/1414-431X202010717

**Published:** 2021-04-02

**Authors:** Qizuan Chen, Pengfan Yang, Qiao Lin, Jiasheng Pei, Yanzeng Jia, Zhonghui Zhong, Shousen Wang

**Affiliations:** 1 Department of Neurosurgery, The 900th Hospital of Joint Logistic Support Force, PLA, Fuzhou, China

**Keywords:** Epilepsy, SVHRP, BDNF, NPY

## Abstract

Scorpion venom is a Chinese medicine for epilepsy treatment, but the underlying mechanism is not clear. Scorpion venom heat-resistant peptide (SVHRP), a peptide isolated from the venom of *Buthus martensii* Karsch, has an anti-epileptic effect by reducing seizure behavior according to a modified Racine scale. The present study aimed to investigate the molecular mechanism of SVHRP on temporal lobe epilepsy. The hippocampus and hippocampal neurons from kainic acid-induced epileptic rats were treated with SVHRP at different doses and duration. Quantitative RT-PCR and immunoblotting were used to detect the expression level of brain-derived neurotrophic factor (BDNF), neuropeptide Y (NPY), cAMP-response element binding protein (CREB), stromal interaction molecule (STIM), and calcium release-activated calcium channel protein 1 (ORAI1). In the hippocampal tissues and primary hippocampal neuron cultures, SVHRP treatment resulted in increased mRNA and protein levels of BDNF and NPY under the epileptic condition. The upregulation of BDNF and NPY expression was positively correlated with the dose level and treatment duration of SVHRP in hippocampal tissues from kainic acid-induced epileptic rats. On the other hand, no significant changes in the levels of CREB, STIM, or ORAI1 were observed. SVHRP may exhibit an anti-epileptic effect by upregulating the expression of BDNF and NPY in the epileptic hippocampus.

## Introduction

Epilepsy is defined as a disorder of the brain characterized by an enduring predisposition to generate epileptic seizures (1). According to public data from the World Health Organization, epilepsy is one of the most common neurological diseases worldwide; there are around 50 million people living with epilepsy (2). A variety of anti-epileptic drugs have been developed and used in first-line therapies for epilepsy treatment (3); however, up to 30% of epilepsy cases can be medically refractory (4).

Scorpion venom is a Chinese ethnomedicine used for the treatment of neuronal diseases, such as apoplexy, cerebral palsy, and epilepsy (5). A recent study demonstrated that scorpion venom heat-resistant peptide (SVHRP), a peptide purified from the venom of *Buthus martensii* Karsch, can reduce seizure susceptibility scored by a modified Racine scale (6). Upon SVHRP treatment, the reduced scores were correlated with the downregulation of proenkephalin mRNA level; however, the detailed molecular mechanism is still unclear. In this study, a kainic acid (KA)-induced rat epilepsy model was used to mimic temporal lobe epilepsy, and the effects of SVHRP on the expression of molecules involved in epileptogenesis, including brain-derived neurotrophic factor (BDNF), neuropeptide Y (NPY), cAMP-response element binding protein (CREB), stromal interaction molecule (STIM), and calcium release-activated calcium channel protein 1 (ORAI1), were analyzed.

## Material and Methods

### Establishment of KA-induced epileptic rats

To establish an epilepsy model in rats, healthy male Sprague-Dawley rats (age: 8-10 weeks; body weight: 200±20 g) purchased from Shanghai SLAC Laboratory Animal Co., Ltd., China (Laboratory animal license number: 42000600018577) were treated with 12 μg/kg of KA (Sigma, USA) by intracerebroventricular injection [lateral ventricle (AP=-0.8 mm, L=+1.5 mm, DV=-3.6 mm)]. In all KA-treated rats, seizures were observed within 1 h. Rats with spontaneous seizures were used for the experiments. The successful induction of epilepsy was confirmed according to the Racine scale (7).

KA-induced epileptic rats were intracerebroventricularly treated with 0.2 or 20 μg/kg of SVHRP, a purified peptide from the venom of the scorpion *Buthus martensii* Karsch obtained from Dalian Medical University, China (National patent number: ZL01106166.9). After 8 h, the hippocampal tissues were collected for further analysis. Normal saline was used as the control.

### Primary hippocampal neuron culture from KA-induced epileptic rats

For primary hippocampal neuron culture, the hippocampal tissues from epileptic rats were collected and cut into slices with 400-600 μm of thickness. Neuronal cells in the entire hippocampal tissues were retrieved by 1 g/L of collagenase (Merck Life Science Co., Ltd., China). The cells were cultured in B27/Neurobasal A with 0.5 mM glutamine, no glutamate, 50 U of penicillin, 0.05 mg/mL of streptomycin, and 5 ng/mL of FGF2 (Thermo Fisher Scientific, USA). After a 10-day culture, the hippocampal neurons were incubated with 0.2 or 20 μg/mL of SVHRP for 1, 8, or 24 h. Cells were then collected for further analysis. To investigate the effects of the dose level of SVHRP, normal saline was used as the control. To investigate the effects of the treatment duration of SVHRP, cells without SVHRP treatment were used as the control.

### Quantitative RT-PCR

Total RNA was extracted from the hippocampal tissues and cultured cells, and cDNA was synthesized by SuperScript III reverse transcriptase (Thermo Fisher Scientific). Quantitative RT-PCR was carried out by Power SYBR GREEN PCR Master mix (Thermo Fisher Scientific) and Applied Biosystems™ 7500 Real-Time PCR system (Thermo Fisher Scientific). Primer sets used for detecting the mRNA expression level of BDNF, NPY, CREB, STIM, ORAI1, and β-actin are listed in Table 1. Relative folds of expression (target gene/β-actin) were calculated using the 2^−ΔΔCT^ method (8).

**Table 1:** Primer sequences for quantitative RT-PCR analysis.

Target	Primer sequence (5′→3′)
BDNF	
Forward	TCAACATAAACCACCAAC
Reverse	TCGCTTCATCTTAGGAGT
NPY	
Forward	GCTTATGACCCGCACTTA
Reverse	TTCAGCCACCCGAGAT
CREB	
Forward	GCACTAAGGTTACAGTGGGAGCAGA
Reverse	ACAGTTCAAGCCCAGCCACAG
STIM	
Forward	GGCTAAGAGAATGGGAAGAATCC
Reverse	CTGGTGGAGAAACTGCCTGAC
ORAI1	
Forward	TGAGGGCGAACAGGTGC
Reverse	GCCAGAGTTACTCCGAGGTGA
β-actin	
Forward	GGAGATTACTGCCCTGGCTCCTA
Reverse	GACTCATCGTACTCCTGCTTGCTG

### Immunoblotting

Protein lysates from the hippocampal tissues and cultured cells were separated by sodium dodecyl sulfate polyacrylamide gel electrophoresis. Proteins were then transferred onto a polyvinylidene difluoride membrane for detection. Antibodies against BDNF, NPY, CREB, p-CREB, STIM, ORAI1, and β-actin were purchased from Abcam (USA).

### Statistical analyses

The non-parametric two-tailed Mann-Whitney test was utilized for statistical comparisons in all data sets. Data in the bar graphs are reported as means±SD. Statistical differences and the number of replicates are reported in each figure.

## Results

**Figure 1:**
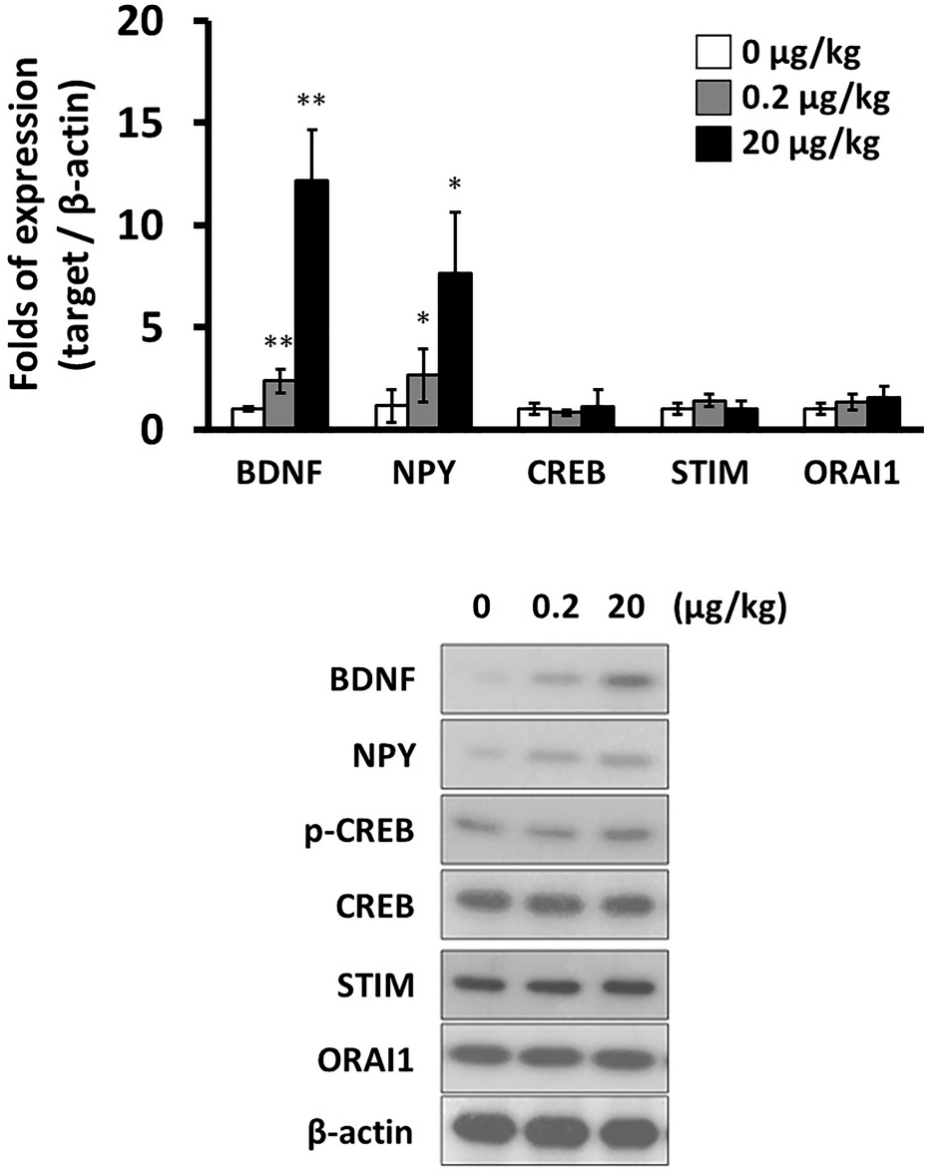
mRNA levels (n=3, **top**) and protein levels (n=3, **bottom**) of brain-derived neurotrophic factor (BDNF), neuropeptide Y (NPY), MP-response element binding protein (CREB), stromal interaction molecule (STIM), and calcium release-activated calcium channel protein 1 (ORAI1) in scorpion venom heat-resistant peptide-treated hippocampal tissues from kainic acid-induced epileptic rats. Data are reported as means±SD. *P<0.05; **P<0.01 (Mann-Whitney test).

**Figure 2:**
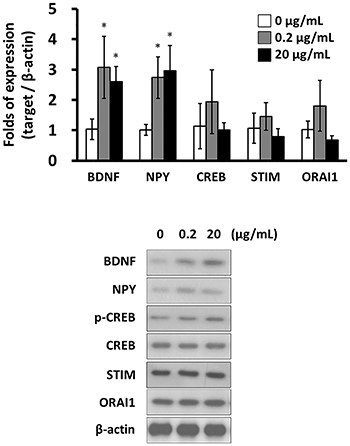
mRNA levels (n=3, **top**) and protein levels (n=3, **bottom**) of brain-derived neurotrophic factor (BDNF), neuropeptide Y (NPY), cAMP-response element binding protein (CREB), stromal interaction molecule (STIM), and calcium release-activated calcium channel protein 1 (ORAI1) in scorpion venom heat-resistant peptide-treated primary hippocampal neuron culture from kainic acid-induced epileptic rats. Data are reported as means±SD. *P<0.05 (Mann-Whitney test).

Of the 20 rats treated with KA for seizure induction, 2 died during the induction of seizures. Successful induction of seizures was observed in 18 rats, which were classified as stage 3 to 5 according to the Racine scale; these rats survived until scheduled euthanasia. In the hippocampal tissues, both mRNA and protein levels of BDNF and NPY were increased by SVHRP. On the other hand, SVHRP did not alter the expression of CREB, STIM, or ORAI1 (Figure 1). Similar mRNA and protein expression patterns with SVHRP treatment were observed in primary hippocampal neuron culture from KA-induced epileptic rats. With SVHRP treatment, the expression of BDNF and NPY was up-regulated, while the expression of CREB, STIM, or ORAI1 was not altered significantly (Figure 2).

**Figure 3:**
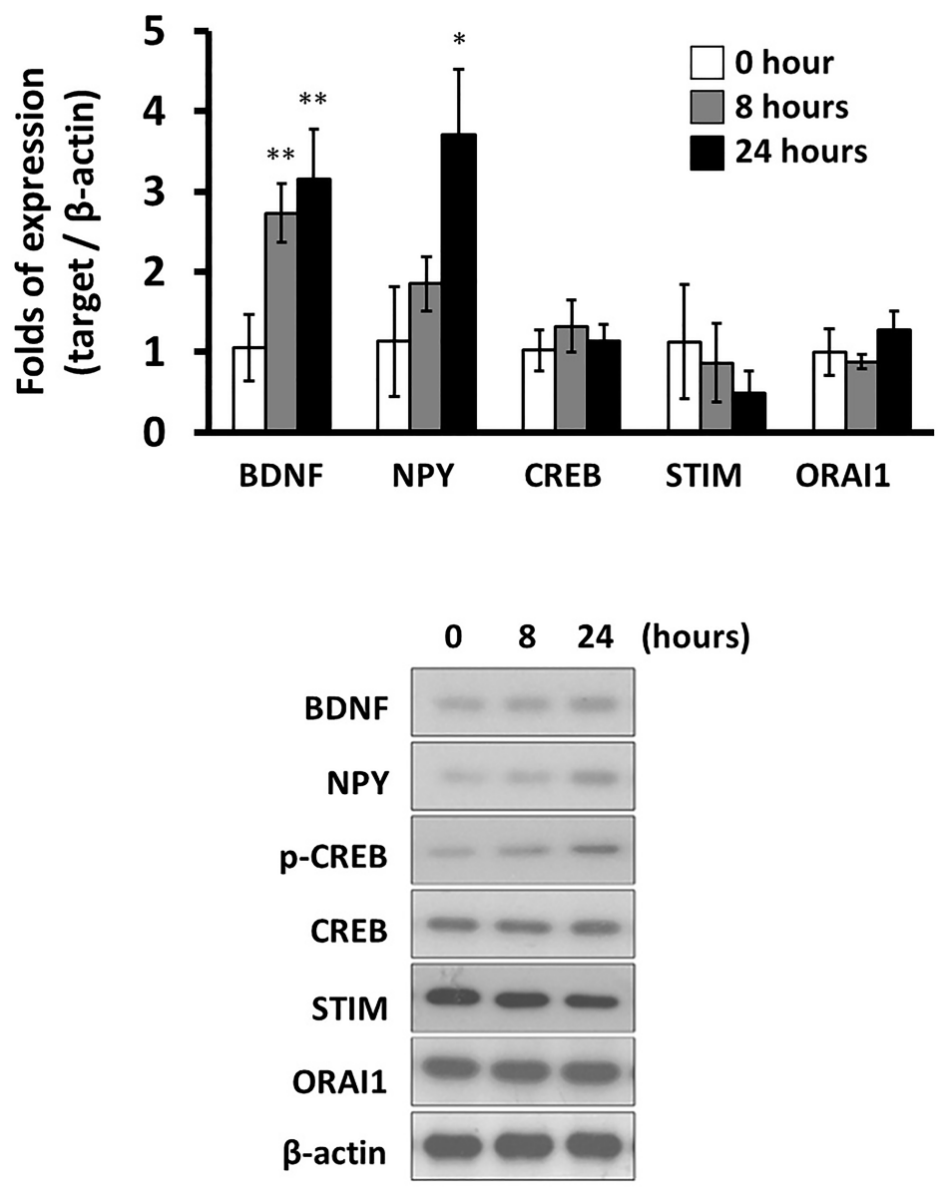
mRNA levels (n=3, **top**) and protein levels (n=3, **bottom**) of brain-derived neurotrophic factor (BDNF), neuropeptide Y (NPY), cAMP-response element binding protein (CREB), stromal interaction molecule (STIM), and calcium release-activated calcium channel protein 1 (ORAI1) in hippocampal tissues from kainic acid-induced epileptic rats with different treatment time of scorpion venom heat-resistant peptide. Data are reported as means±SD. *P<0.05; **P<0.01 (Mann-Whitney test).

The long-term effects of SVHRP on molecules associated with epilepsy were further studied. Eight-hour treatment of SVHRP induced upregulation of BDNF and NPY expression (Figures 1 and 2). This increase was sustained up to 24 h post-treatment at 0.2 μg/kg of SVHRP. On the other hand, the expression of CREB, STIM, or ORAI1 was not significantly changed by long-term treatment of SVHRP (Figure 3).

## Discussion

In this study, SVHRP treatment resulted in the upregulation of BDNF and NPY expression under epileptic condition, while the expression level of the other examined molecules, including CREB, STIM, and ORAI1, were not significantly altered. This differential expression pattern for each epilepsy-associated molecule suggested that the anti-epileptic mechanism of SVHRP may undergo the BDNF-NPY pathway.

BDNF is one of the most studied molecules in the pathophysiology of epilepsy. BDNF increases excitatory neurotransmission in cultured hippocampal neurons (9) and adult hippocampal slices (10). The proepileptogenic effects of BDNF have been systemically reviewed (11,12). Interestingly, seven-day chronic infusion of BDNF into the hippocampus resulted in attenuation of the development of kindling in a rat epilepsy model (13). The anti-epileptogenic effects of BDNF are considered to be achieved by triggering NPY expression (14,15). Upon SVHRP treatment, the mRNA and protein expression levels of BDNF and NPY were positively correlated in the hippocampus and cultured hippocampal neurons from epileptic rats. Moreover, in the hippocampus from epileptic rats, the sustained BDNF expression level triggered NPY expression in a time-dependent manner. These data suggested that SVHRP enhanced BDNF expression and exerted its anti-epileptic effects through BDNF-mediated NPY expression.

CREB has been reported to play a role in transducing neuronal excitatory signals, and decreased CREB levels have been shown to suppress epilepsy (16). Activation of CREB is one of the transcriptional regulatory mechanisms for BDNF and NPY expression (17). In the present study, however, mRNA and protein levels of CREB were not significantly changed upon SVHRP treatment. It is possible that the transcriptional activity of CREB is post-translational regulated (i.e., phosphorylation) and is induced transiently. On the other hand, SVHRP might increase the BDNF and NPY levels through an unexplored CREB-independent transcriptional mechanism. In addition, it is known that aberrant Ca^2+^ flux in the neuron results in epileptic events (18). Activation of an inward Ca^2+^ current in the neuron triggers the initiation of epileptogenic activity (19). STIM and ORAI proteins play a critical role in maintaining cellular Ca^2+^ homeostasis (20). In the present study, the anti-epileptic effects of SVHRP were not achieved by modulating the mRNA and protein levels of STIM and ORAI. In regard to the potential role of SVHRP in Ca^2+^ flux, further studies may focus on the effects of SVHRP on the expression/function of other calcium channels and the formation of STIM-ORAI complexes.

In conclusion, we reported a potential molecular mechanism for the anti-epileptic function of SVHRP. SVHRP upregulated the mRNA and protein levels of BDNF and NPY, providing a potential candidate for epilepsy treatment. Further studies are needed to confirm the correlation between gene/protein expression and changes in electric activity/behavior upon SVHRP treatment.
